# Impact of individualized tidal volume strategies on intraoperative lung protection and inflammatory markers in laparoscopic cholecystectomy: a randomized controlled trial

**DOI:** 10.3389/fphys.2025.1667207

**Published:** 2025-12-01

**Authors:** Xin Wang, Wei Hao, Li-Fang Wu

**Affiliations:** 1 Department of Anesthesiology, The Affiliated Hospital of Inner Mongolia Medical University, Hohhot, China; 2 Department of Anesthesiology, Hohhot First Hospital, Hohhot, China

**Keywords:** HTI56, individualized tidal volume, lung protection, mechanical ventilation, SP-A

## Abstract

**Objective:**

The aim of this study is to evaluate the impact of individualized tidal volume settings, as compared to conventional settings on lung injury in patients undergoing laparoscopic cholecystectomy under mechanical ventilation.

**Methods:**

A total of 40 patients scheduled for elective laparoscopic cholecystectomy at the Affiliated Hospital of Inner Mongolia Medical University between August 2024 and January 2025 were enrolled in this randomized study. Participants were randomly assigned using a random number table to either the control group (Group C, n = 20) or the experimental group (Group T, n = 20), based on the method of tidal volume adjustment. Group C received a conventional tidal volume of 8 mL/kg. In Group T, resting tidal volume was measured preoperatively and used to individualize the mechanical ventilation settings. Serum levels of HTI56, surfactant protein A (SP-A), tumor necrosis factor-α (TNF-α), and interleukin-6 (IL-6) were measured at T1 (prior to anesthesia) and T4 (6 h postoperatively). Mean arterial pressure (MAP) and heart rate were recorded at four time points: T1 (baseline), T2 (20 min post-intubation), T3 (40 min post-intubation), and T4 (6 h postoperatively). Airway parameters including peak airway pressure (Ppeak), plateau pressure (Pplat), and mean airway pressure (Pmean) were documented at T2 and T3.

**Results:**

The average delivered tidal volume in Group T was lower than that in Group C (6.2 ± 0.5 mL/kg vs. 8.0 ± 0.3 mL/kg), indicating a statistically significant difference (*p* < 0.05). At T4, serum levels of IL-6, TNF-α, HTI56, and SP-A were elevated in both groups compared to preoperative values. However, the increases in these inflammatory markers were significantly greater in Group C than in Group T (*p* < 0.05). However, there were no significant differences between the two groups in respiratory mechanics parameters, including Ppeak, Pplat, and Pmean (p > 0.05).

**Conclusion:**

Individualized tidal volume settings were associated with significantly lower postoperative elevations of IL-6, TNF-α, HTI56, and SP-A compared to conventional settings. These findings suggest that tailoring tidal volume based on resting respiratory parameters may help mitigate lung capillary barrier injury by attenuating the inflammatory response, thereby enhancing pulmonary protection during mechanical ventilation.

## 1 Introduction

During laparoscopic cholecystectomy, mechanical ventilation serves as a critical component of respiratory support for patients under general anesthesia. However, it is also associated with the risk of ventilator-induced lung injury (VILI), which is frequently mediated by biotrauma (Jaecklin et al., 2015). Biotrauma refers to the mechanical overstretching of lung tissues, which activates cellular mechanoreceptors and stimulates pulmonary inflammatory cells. This initiates a cascade that leads to the excessive release of inflammatory mediators, including the 56 protein of human alveolar type I cells (HTI56), surfactant protein (SP), tumor necrosis factor-alpha (TNF-α), and interleukin-6 (IL-6). These mediators contribute to increased alveolar fluid accumulation and apoptosis of alveolar epithelial cells. Furthermore, they may traverse the compromised pulmonary capillary barrier and enter the systemic circulation, triggering a systemic inflammatory response that can ultimately lead to multiple organ dysfunction syndrome (MODS) (Wang Y. et al., 2022; Wang, 2022).

Findings from previous studies have indicated that mechanical ventilation strategies incorporating low tidal volumes combined with low plateau pressures may reduce the incidence of VILI and improve oxygenation in patients with acute respiratory distress syndrome (ARDS), as well as decrease intraoperative blood loss during laparoscopic liver surgery (Wang L. et al., 2022; Gao et al., 2021). In procedures performed in the prone position under general anesthesia, lung-protective ventilation strategies (LPVS) have been associated with enhanced intraoperative hemodynamic stability and attenuation of pulmonary inflammation, without increasing the incidence of postoperative pulmonary complications. Additional benefits include improved oxygenation, reduced intrapulmonary shunting, decreased postoperative atelectasis, and a lower risk of barotrauma. These strategies have also not been demonstrated to result in excessive carbon dioxide retention, thereby supporting their safety and efficacy in perioperative ventilation management (Xiang, 2023). In the present study, individualized tidal volume settings were determined based on preoperative resting tidal volume measurements and compared with conventional tidal volume settings to assess their influence on lung injury, with the goal of informing clinical ventilation practices. We hypothesize that, compared with the conventional tidal volume setting, individualized tidal volume ventilation based on preoperative resting tidal volume measurements will reduce the postoperative elevations of serum lung injury-related biomarkers (HTI56, SP-A, TNF-α, and IL-6) in patients undergoing laparoscopic cholecystectomy under mechanical ventilation, by mitigating the inflammatory response and alleviating lung capillary barrier injury, thereby achieving better intraoperative lung protection.

## 2 Information and methods

### 2.1 General information

The patients scheduled for elective laparoscopic cholecystectomy at the Affiliated Hospital of Inner Mongolia Medical University between August 2024 and January 2025 were enrolled. The patients ranged in age from 30 to 50 years, with a body mass index (BMI) between 18 and 24 kg/m^2^ and were classified as American Society of Anesthesiologists (ASA) physical status I–II. Surgical duration was limited to within 1.5 h. The sample size was estimated based on the primary outcome indicators of IL-6 levels at 6 h postoperatively, referring to data from a preliminary pilot study and relevant literature (Wang L. et al., 2022; Gao et al., 2021). In our pilot study involving 10 patients (5 per group), the postoperative serum IL-6 level in the conventional tidal volume group (8 mL/kg) was 51.2 ± 3.2 pg/mL, while that in the individualized tidal volume group was 48.5 ± 2.9 pg/mL, with a mean difference of 2.7 pg/mL and a combined standard deviation of 3.05 pg/mL. Using G*Power 3.1 software for calculation, with a significance level (α) of 0.05 (two-tailed), a power (1-β) of 0.8, and a potential dropout rate of 10%, the required minimum sample size was determined to be 18 patients per group. Therefore, we enrolled 20 patients in each group to ensure sufficient statistical power for detecting differences in the primary and secondary outcome indicators.

Exclusion criteria were: (1) hypoxemia (partial pressure of oxygen, PaO_2_ < 60 mmHg), hypercapnia (partial pressure of carbon dioxide, PaCO_2_ > 50 mmHg), or central hypoventilation syndrome; (2) a history of severe arrhythmia, significant cardiac, pulmonary, hepatic, or renal dysfunction, or circulatory instability with decompensated cardiac function; (3) coagulopathy (international normalized ratio [INR] > 1.5); (4) recent or uncontrolled infections or excessive airway mucus secretion; (5) patients assessed preoperatively by anesthesiologists as having difficult airway management or those at high risk for gastric content reflux or aspiration; (6) abnormal airway anatomy, previous tracheotomy, or lobectomy; (7) neurological disorders impairing cooperation; (8) intraoperative blood loss exceeding 1000 mL or vital sign fluctuations beyond the normal range requiring pharmacological intervention.

A total of 55 eligible patients were initially included, while 15 patients were excluded for different reasons, including hypoxemia, hypercapnia, or central hypoventilation syndrome (n = 7), other serious comorbidities (n = 5), recent or uncontrolled infections (n = 2), and abnormal airway anatomy (n = 1). The patient flowchart is shown in Supplementary Figure S1. The finally eligible patients were randomly assigned using a random number table into two groups: the conventional tidal volume group (Group C, n = 20) and the individualized tidal volume group (Group T, n = 20). Ethical approval was obtained from the hospital’s ethics committee. All patients and their families participated voluntarily, after receiving comprehensive information about the intraoperative medications, equipment, and procedures. Written informed consent was obtained from all participants. Given the nature of the intervention—where the experimental group received individualized tidal volume settings based on preoperative resting tidal volume and the control group received a conventional fixed tidal volume of 8 mL/kg—complete blinding of the anesthesiologists responsible for setting and adjusting ventilation parameters was not feasible (i.e., unblinded to the anesthetic team). However, to minimize detection bias, we implemented partial blinding for other key personnel involved in the study: 1) The researchers responsible for collecting and analyzing serum samples (for detecting HTI56, SP-A, TNF-α, and IL-6 levels) and recording observational indicators (such as MAP, HR, and airway pressures) were blinded to the patients’ group assignments; 2) The patients were also blinded to their group allocation, as they were not informed of the specific tidal volume setting details during the perioperative period. This partial blinding strategy was designed to reduce potential bias in data collection and outcome assessment.

### 2.2 Anesthesia methods

All patients in both groups followed standard preoperative fasting and fluid restriction protocols. Patients in Group T underwent pulmonary function testing prior to surgery to determine resting tidal volume. Upon entering the operating room, patients were positioned supine. Preoperative education was provided to alleviate anxiety and promote patient cooperation during anesthesia and surgery. Routine monitoring included five-lead electrocardiogram (ECG), non-invasive blood pressure (NBP), peripheral oxygen saturation (SpO_2_), heart rate (HR), and bispectral index (BIS). Peripheral venous access was established in the upper limb, and intravenous infusion of sodium lactate Ringer’s solution was initiated. Anesthesia induction was achieved using intravenous sufentanil (0.4 μg/kg), etomidate (0.3 mg/kg), and rocuronium bromide (0.6 mg/kg). Endotracheal intubation was performed once BIS values reached the target range of 40–60. Proper placement of the endotracheal tube was confirmed and connected to the anesthesia machine for end-tidal carbon dioxide (PetCO_2_) monitoring. Ventilation settings included an inspired oxygen concentration of 50%, oxygen flow rate of 1 L/min, inspiratory-to-expiratory ratio (I:E) of 1:2, and peak airway pressure maintained below 30 cmH_2_O. Patients in Group C received a conventional tidal volume of 8 mL/kg with an initial respiratory rate of 12 breaths per minute. Group T received an individualized tidal volume based on preoperative resting tidal volume, also with an initial respiratory rate of 12 breaths per minute. The respiratory rate was adjusted as needed to maintain PetCO_2_ between 35 and 45 mmHg. Positive end-expiratory pressure (PEEP) was continuously applied to both groups of patients from the start of mechanical ventilation until the end of the surgery, with a set value of 5 cmH_2_O. To ensure the strict standardization of PEEP between the two groups, during the operation, the respiratory rate was adjusted only based on the PetCO_2_ and the results of arterial blood gas analysis. The PEEP level remained unchanged throughout the operation and was not adjusted due to other factors such as changes in the patient’s position and blood pressure fluctuations. The detailed ventilator settings were presented in the Supplementary Table S1. Intraoperative anesthesia maintenance included continuous intravenous infusion of propofol and remifentanil, titrated according to BIS values and hemodynamic parameters. Body temperature was monitored intermittently and maintained within 35.5 °C–36.5 °C by adjusting the ambient temperature. Blood pressure was maintained within ±20% of the baseline value, and HR was maintained between 60 and 100 beats per minute. At the conclusion of surgery, 0.5% ropivacaine was administered at the incision site for local anesthesia. Extubation was performed once standard extubation criteria were met, followed by a 5-min observation period prior to transfer to the post-anesthesia care unit (PACU).

### 2.3 Observational indicators

Heart rate and mean arterial pressure (MAP) were recorded at four time points: T1 (before anesthesia), T2 (20 min after intubation), T3 (40 min after intubation), and T4 (6 h postoperatively). Additional recorded parameters included sex, age, BMI, ASA classification, duration of anesthesia, surgical time, time to PACU entry, and ventilatory pressures (peak airway pressure [Ppeak], plateau pressure [Pplat], and mean airway pressure [Pmean]) at T2 and T3.

### 2.4 Laboratory indicators

At T1 and T4, 5 mL of peripheral venous blood was collected from each patient. Samples were allowed to clot at room temperature for 10–20 min, followed by centrifugation at 3000 rpm for 10 min. The supernatant was separated and stored in cryogenic vials at −80 °C. Serum levels of IL-6, TNF-α, HTI56, and surfactant protein A (SP-A) were measured using enzyme-linked immunosorbent assay (ELISA).

### 2.5 Statistical analysis

Statistical analyses were conducted using SPSS version 23.0. All continuous variables for baseline characteristics (e.g., age, BMI, anesthesia time, surgery time) were tested for normality using the Shapiro–Wilk test before statistical analysis. The results of the normality test confirmed that these continuous variables all conformed to a normal distribution (all p > 0.05). Therefore, they were presented as mean ± standard deviation (±SD) and analyzed using independent sample t-tests for intergroup comparisons. The paired t-tests were used to compare the serum levels of inflammatory markers between preoperative time point and postoperatively time point within each group. For categorical variables (e.g., sex, ASA classification), they were expressed as frequency and percentage (n/%) and analyzed using the chi-square test. Repeated measures analysis of variance was used to assess changes over time. Intergroup comparisons were made using independent sample *t*-tests or chi-square tests, as appropriate. A significance level of α = 0.05 was adopted, and *p* values less than 0.05 were considered statistically significant.

## 3 Results

### 3.1 Comparison of general characteristics between the two groups

No statistically significant differences were observed between the two groups with respect to sex, age, or BMI (*p* > 0.05) (Table 1).

**TABLE 1:** Baseline characteristics of patients in the two groups [ $\bar{X}$ ±SD, n (%)].

Indicator	Control group (n = 20)	Trial group (n = 20)	χ^2^/t	P
Sex (male)	9 (45%)	8 (40%)	0.102	0.749
Age (years)	45.25 ± 3.57	45.15 ± 3.68	0.087	0.931
BMI(kg/m^2^)	22.42 ± 1.30	22.32 ± 1.27	0.234	0.816
ASA (Class II)	17 (85%)	16 (80%)	0.173	0.677
Anesthesia time (min)	71.15 ± 2.80	71.25 ± 3.32	−0.103	0.919
Surgery time (min)	67.1 ± 2.92	67.3 ± 3.05	−0.212	0.833
Time to PACU (min)	34.8 ± 2.93	34.9 ± 2.79	−0.111	0.913

Continuous variables (age, BMI, anesthesia time, surgery time) were tested for normality via the Shapiro–Wilk test and confirmed to be normally distributed (*p* > 0.05); categorical variables (sex, ASA, classification) were analyzed using the chi-square test.

### 3.2 Comparison of respiratory mechanics and hemodynamic parameters between the two groups

**FIGURE 1:**
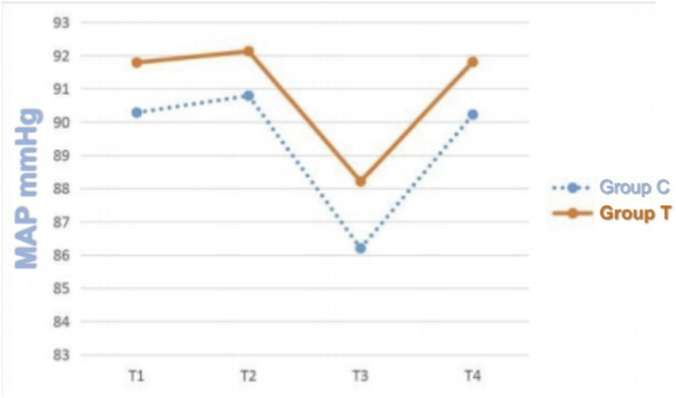
MAP trends at T1, T2, T3, and T4 in the two groups. Note: T1 = before anesthesia; T2 = 20 min after intubation; T3 = 40 min after intubation; T4 = 6 h after surgery.

**FIGURE 2:**
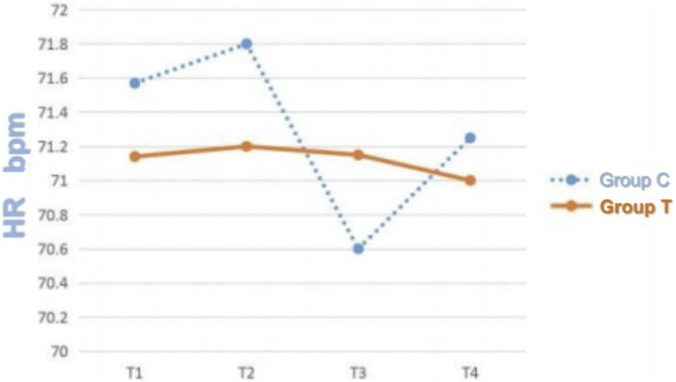
HR Trends at T1, T2, T3, and T4 in the Two Groups. Note: T1 = before anesthesia; T2 = 20 min after intubation; T3 = 40 min after intubation; T4 = 6 h after surgery.

The average delivered tidal volume in Group T was 6.2 ± 0.5 mL/kg, compared to 8.0 ± 0.3 mL/kg in Group C, confirming a statistically significant separation (*p* < 0.05). No significant differences were detected between the two groups in respiratory mechanics parameters, including Ppeak, Pplat, and Pmean (*p* > 0.05) (Table 2). Similarly, there were no statistically significant differences in hemodynamic parameters, specifically MAP and HR, at the recorded time points (*p* > 0.05) (Figures 1, 2). Repeated-measures ANOVA showed no significant within-group × between-group interaction effect on MAP (F = 0.82, p = 0.41), no significant between-group effect (F = 0.35, p = 0.56), and a significant within-group effect (F = 4.21, p = 0.02), indicating that MAP changed significantly over time in both groups but with no differential change patterns between groups. For Ppeak, repeated-measures ANOVA revealed no significant within-group effect (F = 1.15, p = 0.29), between-group effect (F = 0.04, p = 0.84), or within-group × between-group interaction effect (F = 0.12, p = 0.73), confirming stable Ppeak levels across time points and no differences between groups.

**TABLE 2:** Respiratory mechanics parameters at T2 and T3 in the two groups ( $\bar{X}$ ± SD).

Indicator	Time point	Control group (n = 20)	Trial group (n = 20)	P
Ppeak (cmH_2_O)	T2	12.35 ± 0.88	12.40 ± 0.88	0.858
	T3	12.15 ± 0.67	12.25 ± 0.85	0.682
Pplat (cmH_2_O)	T2	9.60 ± 1.10	9.55 ± 0.83	0.871
	T3	9.70 ± 0.80	9.80 ± 0.77	0.689
Pmean (cmH_2_O)	T2	4.60 ± 0.68	4.40 ± 0.50	0.297
	T3	4.65 ± 0.59	4.75 ± 0.72	0.632

T2 = 20 min after intubation; T3 = 40 min after intubation.

### 3.3 Comparison of preoperative and postoperative serum inflammatory markers between the two groups

In both groups, postoperative serum concentrations of IL-6, TNF-α, HTI56, and SP-A were significantly elevated compared to preoperative values (*p* < 0.05). No statistically significant differences in these inflammatory markers were observed between the groups at T1 (*p* > 0.05). However, at T4, significantly lower levels of IL-6, TNF-α, HTI56, and SP-A were recorded in the individualized tidal volume group (Group T) compared to the conventional group (Group C) (*p* < 0.05), indicating that individualized tidal volume settings were associated with a reduction in postoperative inflammatory response (Table 3).

**TABLE 3:** Serum inflammatory marker levels before and after surgery in the two groups ( $\bar{X}$ ±SD).

Indicator	Time point	Control group (n = 20)	Trial group (n = 20)	MD	P
SP-A (pg/mL)	T1	88.76 ± 6.00	89.41 ± 5.56	−0.648	0.725
	T4	118.8 ± 9.64^#^	110.99 ± 8.67#	7.803	0.011^*^
HTI56(pg/mL)	T1	237.79 ± 46.97	238.34 ± 7.56	−0.555	0.959
	T4	268.5 ± 37.82^#^	245.78 ± 26.74#	22.721	0.034^*^
IL-6 (pg/mL)	T1	38.67 ± 3.38	39.64 ± 2.18	−0.968	0.288
	T4	51.11 ± 3.19^#^	48.68 ± 2.80#	2.433	0.014^*^
TNF-α(pg/mL)	T1	43.83 ± 3.51	43.18 ± 5.76	0.644	0.672
	T4	61.35 ± 5.58^#^	56.12 ± 7.33#	5.224	0.015^*^

T1 = before anesthesia; T2 = 20 min after intubation; T3 = 40 min after intubation; T4 = 6 h after surgery. Compared with T1, #*p* < 0.05; MD = $\bar{X}$ Control group – $\bar{X}$ Trial group, *p < 0.05.

## 4 Discussion

Laparoscopic hepatobiliary surgery has become increasingly prevalent due to its broad clinical indications, minimally invasive nature, shorter hospitalization periods, and its effectiveness in reducing both surgical stress and postoperative complications (Yeh et al., 2020). General anesthesia with mechanical ventilation is commonly employed during such procedures. Perioperatively, administration of high-concentration oxygen during mechanical ventilation is standard practice. This approach increases oxygen reserves during induction and emergence, prolongs the safe apneic interval for tracheal intubation, enhances hypoxia tolerance, and decreases the risk of postoperative nausea, vomiting, and surgical site infections. However, the routine use of high-concentration oxygen has been associated with the development of atelectasis and the exacerbation of VILI. These effects may contribute to increased intrapulmonary shunting and impaired pulmonary function, thereby elevating the risk of both intraoperative and postoperative pulmonary complications.

In recent years, increasing recognition has been given (both nationally and internationally) to the importance of individualized mechanical ventilation strategies. It is widely accepted that ventilation parameters must be tailored to the pulmonary physiological characteristics of each patient, with dynamic adjustments to optimize lung stability and prevent alveolar collapse. Individualized mechanical ventilation, when implemented during general anesthesia, facilitates improved lung compliance, minimizes carbon dioxide retention, optimizes tidal compliance, and stabilizes both plateau airway pressure and dead space volume (Battaglini et al., 2021). These outcomes collectively promote alveolar recruitment and reduce the risk of VILI. Furthermore, individualized strategies have been associated with a reduction in the systemic release of inflammatory mediators, supporting their clinical applicability. In the present study, no significant differences were observed between the groups in terms of anesthesia duration, surgical duration, or time to transfer to the PACU, indicating that both individualized and conventional tidal volume settings did not adversely affect intraoperative anesthesia or surgical workflow. Additionally, the lack of statistically significant differences in respiratory mechanics and hemodynamic parameters between the two groups suggests that both ventilation strategies effectively maintained adequate oxygenation and circulatory stability during mechanical ventilation under general anesthesia with endotracheal intubation.

Mechanical ventilation has been identified as a contributing factor to the development of postoperative pulmonary complications, including pneumonia, atelectasis, and respiratory failure (Li et al., 2024). These complications may result in postoperative hypoxemia and hypotension, thereby exacerbating ischemic and hypoxic conditions at surgical anastomoses. Furthermore, these conditions stimulate the systemic release of inflammatory markers, including HTI56, SP, TNF-α, and IL-6 (Wang et al., 2024). Alveolar type I cells cover over 95% of the alveolar surface. Injury to these cells can lead to membrane blebbing and detachment into the alveolar lumen, contributing to the appearance of HTI56 in the peripheral circulation. As HTI56 has not been detected in extrapulmonary tissues, its presence in serum is considered a specific marker of pulmonary parenchymal injury and may hold diagnostic value for assessing lung tissue damage (Dobbs et al., 1999). Pulmonary surfactant is a lipid–protein–carbohydrate complex secreted by alveolar type II cells that lines the alveolar epithelium. Approximately 8% of its composition comprises SP, which bind specifically to phospholipids. These proteins are critical for maintaining alveolar surface tension and pulmonary homeostasis and serve as biomarkers for pulmonary vascular permeability (Retamal et al., 2021).

Among the four surfactant protein subtypes, SP-A is the most abundant, comprising approximately 50% of the total SP content, and is the earliest identified and most strongly expressed subtype. In this study, postoperative serum concentrations of IL-6, TNF-α, HTI56, and SP-A were elevated in both groups relative to preoperative values, indicating an increase in systemic inflammatory activity following surgery. These findings are supported by experimental data reported by Shi et al., which demonstrated that pulmonary contusion and hypoxia result in direct injury to alveolar type II cells, subsequently impairing surfactant production. These conditions also damage the pulmonary vascular endothelial cells and disrupt the alveolar-capillary barrier, resulting in the release of SP-A into the circulation and elevated serum SP-A levels, which aligns with the trends observed in the current findings (Wang, 2022).

TNF-α is recognized as a sensitive biomarker that reflects the extent of tissue injury and is among the earliest proinflammatory cytokines to appear following trauma. Its serum concentration has been demonstrated to correlate positively with the severity of tissue damage (Shaw et al., 2023). As a principal mediator of the inflammatory response, TNF-α is often produced in conjunction with IL-6 and interleukin-8 (IL-8). TNF-α contributes to pulmonary injury by directly damaging alveolar endothelial cells, activating polymorphonuclear leukocytes, and impairing surfactant production. These mechanisms underscore its critical role in the initiation and progression of both intraoperative and postoperative lung injury. Given these effects, reducing inflammatory cell aggregation and inhibiting the release of proinflammatory mediators has become a priority in the clinical management of mechanically ventilated patients (Zhang, 2023; Bailly, 2022).

In this study, no statistically significant differences were observed between the experimental and control groups in serum concentrations of IL-6, TNF-α, HTI56, and SP-A at T1. However, significant differences were noted at T4, with lower levels recorded in the individualized tidal volume group. These findings indicate that tidal volume settings influence the magnitude of the postoperative inflammatory response. and that individualized tidal volume was associated with a reduction in these inflammatory markers at T4. This reduction supports the protective role of individualized ventilation strategies in mitigating lung injury. Previous animal model research by Huang Jinshui demonstrated that low tidal volume ventilation attenuated lung injury in rats exposed to 4-nitroquinoline-1-oxide (4NQO) via drinking water. Moreover, the application of low tidal volume in combination with low PEEP and lung recruitment maneuvers significantly reduced IL-6 and TNF-α concentrations in bronchoalveolar lavage fluid among male Sprague–Dawley rats (Shaw et al., 2023).

SP-A has been identified as a key biomarker reflecting the severity of acute lung injury. It is essential for preserving pulmonary homeostasis and plays a crucial role in maintaining the integrity of the alveolar-capillary barrier and normal respiratory function (Wei et al., 2023). In cases of acute lung injury, large quantities of SP-A may enter the systemic circulation, resulting in elevated serum SP-A concentrations that are closely associated with both the onset and progression of pulmonary damage. In the current study, serum SP-A concentrations were significantly lower in the individualized tidal volume group compared to the control group at T4, further substantiating the lung-protective effect associated with individualized tidal volume strategies.

A key consideration regarding our findings is the discrepancy between nonsignificant driving pressure differences (derived from similar plateau pressure and consistent 5 cmH_2_O PEEP across groups) and clear intergroup disparities in lung injury/inflammation markers. This can be partially explained by the limitations of driving pressure—while it reflects global lung mechanical load, it fails to capture regional variations: the conventional 8 mL/kg tidal volume may have caused regional overdistension in less compliant lung areas, triggering local inflammation and epithelial damage, whereas individualized tidal volumes (6.2 ± 0.5 mL/kg) optimized regional ventilation to mitigate this, even without altering global driving pressure. It should be acknowledged that unmeasured factors (e.g., regional lung mechanics, quantifiable atelectasis, subtle baseline pulmonary differences) could also contribute, as we lacked direct assessments of atelectasis or regional compliance, though stable hemodynamics and consistent PEEP suggest minimal severe atelectasis impact. These points imply our biomarker findings may stem not just from global mechanical differences but regional optimization, highlighting that individualized tidal volumes offer lung protection without compromising global respiratory mechanics—yet future studies with regional monitoring are needed to clarify these interactions and strengthen causal links.

It was undeniable that this study had several limitations. Although this study managed to control the differences in lung function between groups as much as possible by strictly incorporating criteria (such as BMI 18–24 kg/m^2^, ASA grade I-II, and no underlying cardiopulmonary diseases), it was still impossible to completely rule out the interference of potential heterogeneity in the baseline lung mechanical state on the research results, which may affect the rigor of the comparison of ventilation parameters and inflammatory indicators between groups. Moreover, as the initial design of this study focused on the impact of tidal volume on inflammatory markers related to lung injury, clinical outcome indicators such as postoperative pulmonary complications (such as pneumonia and atelectasis), additional oxygen therapy requirements, length of hospital stay, and postoperative ICU transfer rate were not included. Furthermore, the study also had limitations including a small sample size (40 patients total, 20 per group) that may restrict detecting subtle secondary outcome differences and raise type II error risk despite statistical power calculations, and a single-center design with homogeneous inclusion criteria that ensured internal validity but undermines external validity as results may not apply to diverse patient groups or other laparoscopic surgeries. Additionally, the study focused on the association between individualized tidal volume and biomarker/clinical outcome changes but lacked mechanistic research (e.g., alveolar lavage fluid analysis, molecular pathway exploration), limiting explanations for observed phenomena and establishment of a direct causal link between the ventilation strategy and lung protective effects. Also, while this study focused on inflammatory biomarkers and core respiratory/hemodynamic parameters, this study did not explicitly measure compliance or include imaging or surrogate markers to quantify atelectasis. To address this mechanistic gap, future studies will incorporate direct compliance monitoring and atelectasis-focused assessments to clarify how individualized tidal volume interacts with lung compliance and atelectasis formation, thereby strengthening the causal link between ventilation strategy, lung mechanics, and inflammatory outcomes.

## 5 Conclusion

The overall findings of this study indicate that individualized tidal volume settings during anesthesia effectively maintain stable respiratory mechanics and hemodynamic parameters. Compared to conventional tidal volume ventilation, individualized ventilation significantly reduced postoperative levels of IL-6, TNF-α, HTI56, and SP-A, thereby limiting inflammation-induced injury to the alveolar-capillary interface and contributing to lung protection.

## Data Availability

The raw data supporting the conclusions of this article will be made available by the authors, without undue reservation.

## Supplementary material

SUPPLEMENTARY FIGURE S1 CONSORT flow diagram.

## Abbreviations

VILI, Ventilator-induced lung injury; HTI 56, 56protein of Human Alveolar Type I Cells; SP, Surfactant Protein; TNF-α, Tumor Necrosis Factor-alpha; IL-6, Interleukin-6; ARDS, acute respiratory distress syndrome; MAP, mean arterial pressure; HR, heart rate; Ppeak, peak airway pressure; Pplat, plateau pressure; Pmean, mean airway pressure; LPVS, Lung protective ventilation strategy; BMI, body mass index; ASA American society of anesthesiologists; PaO_2_, arterial partial pressure of oxygen; PaCO_2_, arterial partial pressure of carbon dioxide; INR, international normalized ratio; ECG, electrocardiogram; ECG; NBP, noninvasive blood pressure; SpO_2_, saturation of blood oxygen; BIS, bispectral index; PetCO_2_, end-tidal carbon dioxide partial pressure; PACU, Post Anesthesia Care Unit; ELISA, enzyme-linked immunosorbent assay; IL-8, interleukin-8, IL-8; PEEP, positive end expiratory pressure.
