# Supplementary material for: Impact of individualized tidal volume strategies on intraoperative lung protection and inflammatory markers in laparoscopic cholecystectomy: a randomized controlled trial
Source: Front Physiol. 2025 Dec 1;16:1667207. doi: 10.3389/fphys.2025.1667207 (PMC12702722; doi:10.3389/fphys.2025.1667207)
Supplement: Supplementary file 1 [file Table1.docx]

Supplementary Table 1. The details of ventilator settings.

| Items | Settings |
| --- | --- |
| An inspired oxygen concentration | 50% |
| Oxygen flow rate | 1 L/min |
| Inspiratory-to-expiratory ratio (I:E) | 1:2 |
| Peak airway pressure | below 30 cmH₂O |
| Respiratory rate | an initial respiratory rate of 12 breaths per minute and adjusted as needed to maintain PetCO₂ between 35 and 45 mmHg |
| Positive end-expiratory pressure (PEEP) | 5 cmH₂O |
