# Supplementary figures and images for: Impact of individualized tidal volume strategies on intraoperative lung protection and inflammatory markers in laparoscopic cholecystectomy: a randomized controlled trial
Source: Front Physiol. 2025 Dec 1;16:1667207. doi: 10.3389/fphys.2025.1667207 (PMC12702722; doi:10.3389/fphys.2025.1667207)

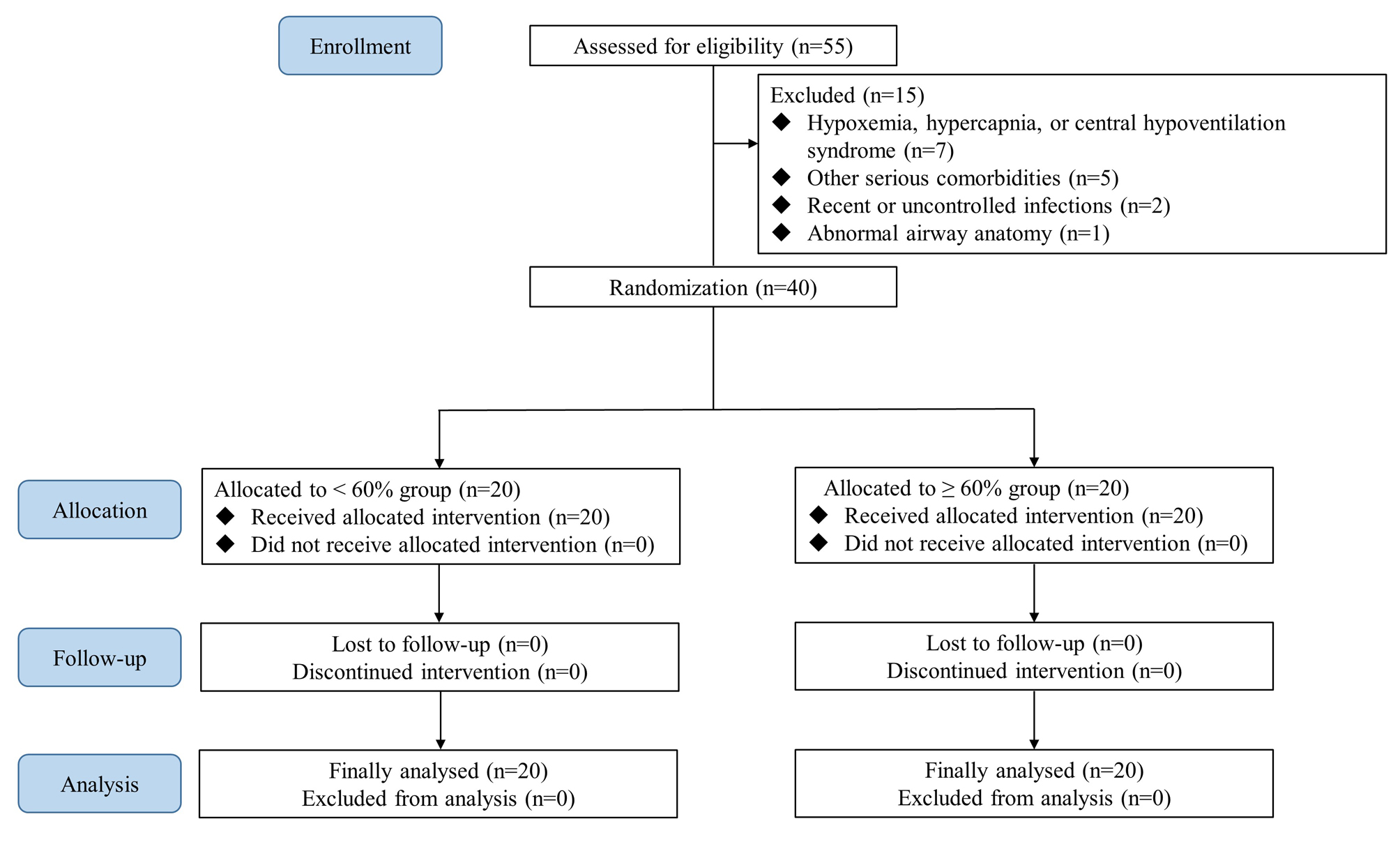

Supplement: Supplementary file 2 [file Image1.jpeg]
